# Local antibiotic treatment with calcium sulfate as carrier material improves the outcome of debridement, antibiotics, and implant retention procedures for periprosthetic joint infections after hip arthroplasty – a retrospective study

**DOI:** 10.5194/jbji-7-11-2022

**Published:** 2022-01-20

**Authors:** Katharina Reinisch, Michel Schläppi, Christoph Meier, Peter Wahl

**Affiliations:** Division of Orthopaedics and Traumatology, Cantonal Hospital Winterthur, Winterthur, Switzerland

## Abstract

**Purpose**: Debridement, antibiotics, and implant retention (DAIR) is an established treatment modality in periprosthetic joint infections (PJIs), but success rates vary. This study compared the success of DAIR for PJIs after a total hip arthroplasty (THA), with or without local antibiotic delivery with CaSO ${}_{4}$ as the carrier material. **Methods**: A retrospective review of DAIR for PJIs after THA performed between 2010 and 2018, including 41 patients is conducted. A total of 27 patients were treated by DAIR with local antibiotics with CaSO ${}_{4}$ as the carrier material, and 14 patients were treated by a standard DAIR. The endpoints were treatment failure, defined as the need for a reoperation, either a second DAIR or a prosthesis removal or exchange due to persistent or recurrent infection, the initiation of a long-term suppressive antibiotic treatment, or death related to infection. **Results**: Considering any reoperation as an outcome, 11 of 14 cases treated without AB-CaSO ${}_{4}$ (79 %) and 4 of the 27 cases treated with AB-CaSO ${}_{4}$ failed (15 %). Considering revision as an outcome, 9 out of 14 cases treated without AB-CaSO ${}_{4}$ (64 %) and 4 of the 27 cases treated with AB-CaSO ${}_{4}$ (15 %) failed. A Kaplan–Meier survival analysis showed that local antibiotic delivery with CaSO ${}_{4}$ as the carrier material led to a significantly longer infection-free survival, considering any surgical revision ( p<0.0001 ; hazard ratio 8.9 (95 % CI 2.8–28.2)) or revision with component exchange ( p=0.0015 ; hazard ratio 5.6 (95 % CI 1.7–18.2)) as the endpoint. **Conclusion**: The addition of local antibiotics with CaSO ${}_{4}$ as the carrier material to DAIR for PJIs after THA significantly increases success rates, such as infection-free survival, any reoperation, and revision with component exchange in particular.

## 1 Introduction

Periprosthetic joint infection (PJI) has an incidence of approximately 1 % after total hip arthroplasty (THA) and 2 % after total knee arthroplasty (TKA) in large register-based studies (Kurtz et al., 2010; Gundtoft et al., 2015; Huotari et al., 2015). PJI is associated with high morbidity and mortality (Kurtz et al., 2012; Webb et al., 2014; Lum et al., 2018; Natsuhara et al., 2019) and represents a high economic and logistic burden on the healthcare system (Kurtz et al., 2012; Vanhegan et al., 2012; Haddad et al., 2017; Fischbacher et al., 2018; Sousa et al., 2018; Schwartz et al., 2020).

A two-stage exchange remains the standard therapy for PJIs (Cooper and Della Valle, 2013; Osmon et al., 2013). While being very successful regarding the eradication of infection, this option is associated with functional outcomes that are worse (Oussedik et al., 2010; Dzaja et al., 2015; Grammatopoulos et al., 2017a, b; Herman et al., 2017), with higher complications and increased mortality rates compared to implant-retaining procedures and one-stage exchange (Berend et al., 2013; Browne et al., 2017; Barton et al., 2020). PJIs may also be treated successfully by debridement, antibiotics, and implant retention (DAIR) if the implant is well-fixated and if biofilm-active antibiotic treatment is available (Trebse et al., 2005; Osmon et al., 2013; Grammatopoulos et al., 2017b). The reported success rates of DAIR, regarding the eradication of infection, vary from 35 % to 88 % (Deirmengian et al., 2003; Trebse et al., 2005; Marculescu et al., 2006; Vilchez et al., 2011; Font-Vizcarra et al., 2012; Aboltins et al., 2013; Lora-Tamayo, 2013; Grammatopoulos et al., 2017b; Lora-Tamayo et al., 2017). The variability in the results may be explained by the heterogeneity of the cohorts, the influence of the duration of the symptoms and of the causative microorganisms, variable definitions of success, and differing follow-up periods. Even a hard outcome, such as reoperation, may result in differences in analysis, as the persistence of infection is not necessarily diagnosed or reoperated and a suppressive antibiotic treatment is possibly started instead (Prendki et al., 2017; Sandiford et al., 2020). Repeated DAIR procedures may also be successful, with persistence of infection after a first DAIR not necessarily requiring component removal (Grammatopoulos et al., 2017b; Wouthuyzen-Bakker et al., 2020). Nevertheless, the functional outcomes of successful DAIR correspond to results obtained after primary THA or TKA without PJI (Barros et al., 2019), which are much better than the functional outcomes after two-stage exchange (Dzaja et al., 2015; Grammatopoulos et al., 2017a; Herman et al., 2017).

The efficacy of the antibiotic treatment, and thus the success rate of DAIR, may potentially be increased with local antibiotic delivery. Calcium sulfate (CaSO ${}_{4})$ is a particularly interesting carrier material for this indication. As it dissolves, it does not require secondary removal as bone cement (polymethyl methacrylate – PMMA) does (McKee et al., 2010). It is soft and does not cause relevant third-body wear on prosthetic components (Heuberger et al., 2014), and it is compatible with many antibiotics (Wahl et al., 2018). Biofilm-active concentrations of vancomycin may be obtained locally for at least 2 weeks and with therapeutic release for some months (Post et al., 2017; Wahl et al., 2017; Baeza et al., 2019). The aim of this study was to compare the success rates of DAIR for PJI after THA with or without local antibiotic delivery with CaSO ${}_{4}$ as the carrier material.

**Figure 1:**
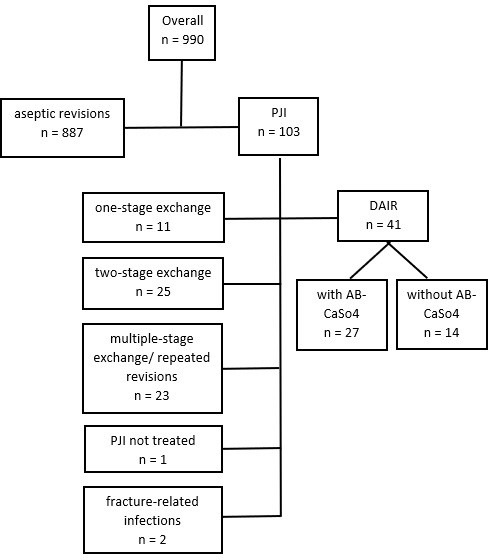
Flowchart showing the process of patient identification, exclusion, and grouping of PJI treatment options. Repeated revisions were excluded with regard to higher failure rates. Only PJIs following primary THA were considered.

## 2 Patients and methods

A retrospective, single-institution study of patients treated with DAIR between January 2010 and December 2018 for PJIs after THA was conducted. Cases were identified by reviewing the in-house coding database for codes potentially used for the revision of THA (Swiss Operation CHOP codes 00.70 to 00.79 and 81.52.00 to 81.53.99) and reviewing the prospectively collected database of patients receiving local antibiotic treatment with CaSO ${}_{4}$ as the carrier material. DAIR procedures performed on primary THA, excluding larger-sized revision implants, were manually identified. Cross-verification was performed with data from the Swiss Arthroplasty Register (SIRIS) to identify septic revisions performed after in-house primary THA. However, the latter identified no supplementary revisions. Thus, 990 revision THAs were identified, of which 41 DAIR procedures could be analyzed for this study (Fig. 1). Demographic data, comorbidities, surgical data, infection characteristics (including diagnostic criteria, type of microorganism, and antibiotics administered), and follow-up data were then retrieved from the electronic patient files.

The diagnosis of PJIs is based on current international guidelines (Zimmerli et al., 2004; Osmon et al., 2013). At least one of the two attending physicians of the hip team were involved in all cases, with one physician being replaced in 2015. Since 2010, an anterior approach, performed in supine position on a traction hemi-table applied to the operated leg, has been the standard in-house procedure for primary THA. Pre-existing, other approaches were also used, which was particularly appropriate for external cases referred for treatment. Debridement repeated within 72 h following the initial DAIR procedure was supposed to be second-look operations as part of the initial treatment plan and was not counted as separate DAIR procedure. While there was no strict rule to perform second-look operations, this was common practice in the early phase of the study. Modular components were always exchanged in DAIR procedures (Svensson et al., 2020) but only once in the case of second-look operations. In case a second look was performed, only the first operation was considered for the Kaplan–Meier analysis. Postoperative antibiotic treatment followed international guidelines, including a biofilm-active drug administered as soon as possible and for a total duration of 12 weeks (Zimmerli et al., 2004; Osmon et al., 2013). Treatment in both groups was otherwise identical, except for the local application of antibiotic-loaded calcium sulfate (AB-CaSO ${}_{4}$ ). AB-CaSO ${}_{4}$ (Osteoset^®^; Wright Medical Technology, Inc., Arlington, Tennessee) was introduced in our institution in 2015 and became routinely applied from 2017 onwards. The technical details regarding preparation of the beads are described elsewhere (Wahl et al., 2017). Routinely, three 25 mL packs of Osteoset^®^ loaded with 2 g vancomycin each were implanted into the hip joint after debridement. This quantity was usually reduced to one or two packs in case of severe renal failure, due to potential for hypercalcaemia. As ceftriaxone causes a major volume expansion, the number of packs, consequently, also had to be reduced as adaptation.

All patients underwent regular clinical and radiologic outpatient follow-up. Since not every patient was able to attend follow-up later than 3 months postoperatively, the patients and/or their general practitioners were contacted by telephone to receive the required information for a follow-up of at least 1 year after the DAIR procedure. Treatment failure was defined as either any reoperation, such as a second DAIR procedure beyond the accepted time limit of 72 h for a second look, or a revision with prosthesis removal or exchange due to persistent or recurrent infection. The introduction of a suppressive antibiotic treatment or death related to infection were also defined as treatment failures. The end of follow-up or death for unrelated causes resulted in censoring.

**Figure 2:**
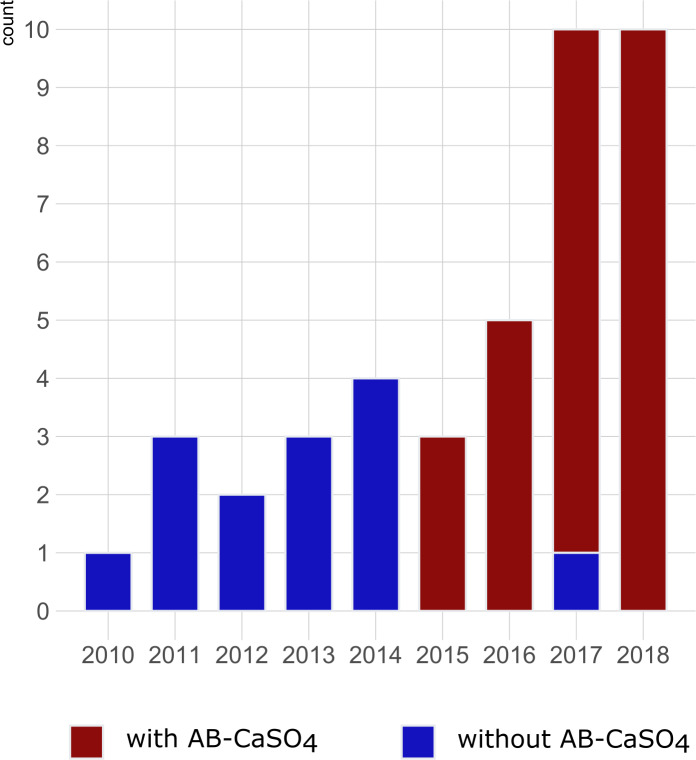
Graphical illustration of the number of cases of DAIR procedures for hip PJIs included per year in the study and color-coded depending on the application of antibiotic-loaded CaSO ${}_{4}$ (AB-CaSO ${}_{4}$ ). AB-CaSO ${}_{4}$ was introduced in our department in 2015. The change happened progressively, with the last DAIR procedure performed without AB-CaSO ${}_{4\;}$ at the beginning of 2017. This does not appear on this figure, as cases operated without AB-CaSO ${}_{4}$ during the transition period fulfilled the exclusion criteria of this study. The increase in cases during the last few years may be indicative of a shift towards DAIR procedures induced by the obviously favorable results with addition of AB-CaSO ${}_{4}$ .

Considering the small number of cases, scalar data are reported with the median and range, and non-parametric tests were used for comparison. Categorical data are reported with number of cases and percentages. Differences in the distribution between groups were analyzed using Fisher's exact test, the chi-squared test for nominal variables, and Wilcoxon's rank sum test for ordinal variables. Kaplan–Meier survival analysis was performed with DAIR or any revision later than 72 h after the first DAIR (Analysis A) and any revision with implant exchange (Analysis B) as endpoints. The subgroup analysis stratified by treatment with and without CaSO ${}_{4}$ was evaluated using a log rank test. Hazard ratios and 95 % confidence intervals were derived from univariate Cox proportional hazards regression models. All analyses were conducted using the software package R (the R Project for Statistical Computing) with a significance level set at p<0.05 .

**Figure 3:**
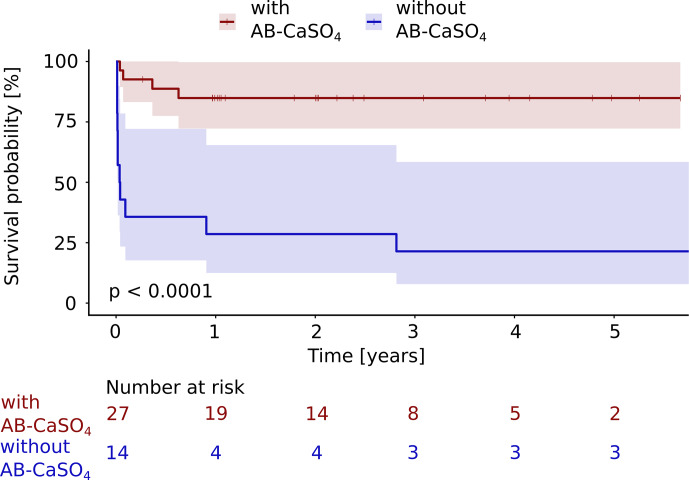
The Kaplan–Meier cumulative survival curves of 41 patients with PJI of the hip treated by DAIR procedure are shown and are stratified by treatment with and without local application of antibiotics with CaSO ${}_{4}$ as the carrier material. The end point was defined as any reoperation, including DAIR or exchange, later than 72 h after the first DAIR. DAIR repeated within 72 h was accepted as a second look and was not counted as a failure. The survival data were compared using the log rank test. A significantly ( p<0.0001 ) longer infection-free cumulative survival could be observed in the intervention group compared to the control group. The confidence interval indicated corresponds to a 95 % confidence interval. The Cox proportional hazard ratio over the whole study period was 8.9 (95 % CI 2.8–28.2) for this analysis, favoring the addition of AB-CaSO ${}_{4}$ .

**Figure 4:**
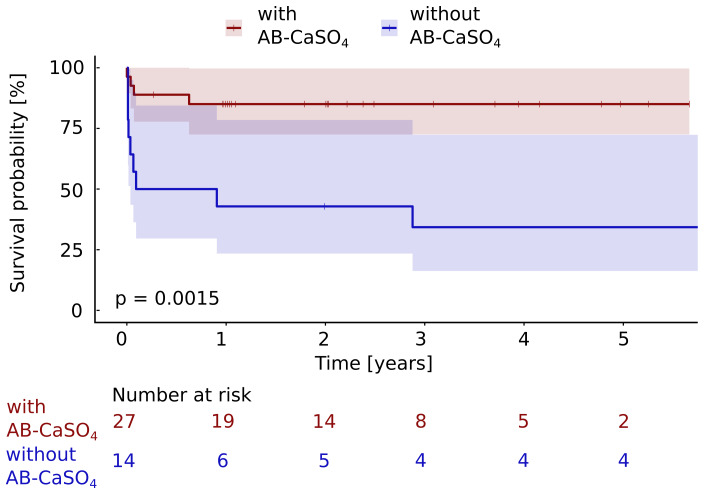
The Kaplan–Meier cumulative survival curves of the 41 patients with PJI of the hip treated by DAIR procedure are shown and are stratified by treatment with and without local application of antibiotics with CaSO ${}_{4}$ as the carrier material. The end point was defined as any reoperation for component exchange. For this analysis, repeat DAIR was not considered as a failure. The survival data were compared using the log rank test. A significantly ( p=0.0015 ) longer infection-free cumulative survival could be observed in the intervention group compared to the control group. The confidence interval indicated corresponds to a 95 % confidence interval. The Cox proportional hazard ratio over the whole study period was 5.6 (95 % CI 1.7–18.2) for a revision with component exchange, favoring the addition of AB-CaSO ${}_{4}$ .

Prior to this study, written general consent for such an analysis was obtained from all patients as a matter of routine. As this study only involved a retrospective review of anonymized data without any intervention for study purposes, Swiss law requires no specific approval by an external ethical committee.

**Table 1:** Demographic data and surgical characteristics of the study population. There are no statistically significant differences between both groups. All categorial data were compared with chi-squared tests, while a non-parametric test was used for age. The resorbable bead kit (RBK) was mixed with vancomycin or ceftriaxone, according to the causative bacteria and identified antibiotic resistances. Osteoset^®^ T (Wright Medical Technology, Inc.) is a ready-to-use product, containing 400 mg of tobramycin sulfate per 10 mL of beads.

	All patients ( n=41 )	DAIR with AB-CaSO ${}_{4}$ ( n=27 )	DAIR without AB-CaSO ${}_{4}$ ( n=14 )	Statistics ( p value)
Age (years), median (range)	70.2 (34.6–91.9)	71.3 (34.6–91.9)	65.3 (51.8–81.5)	
Gender, n (%)				0.67 ${}^{a}$
Male	26 (63.4 %)	16 (59.3 %)	10 (71.4 %)	
Female	15 (36.6 %)	11 (40.7 %)	4 (28.6 %)	
BMI (kg/m ${}^{2}$ ), n (%)				0.21 ${}^{c}$
<24.9	6 (14.6 %)	5 (18.5 %)	2 (14.3 %)	
25.0–29.9	16 (39.0 %)	12 (44.4 %)	4 (28.6 %)	
30.0–34.9	6 (14.6 %)	6 (22.2 %)	0	
35.0–39.9	4 (9.8 %)	2 (7.4 %)	2 (14.3 %)	
>40.0	2 (4.9 %)	2 (7.4 %)	0	
Unknown	6 (14.6 %)	0	6 (42.9 %)	
ASA score n (%)				0.004 ${}^{c}$
1 and 2	17 (41.5 %)	7 (25.9 %)	10 (71.4 %)	
3 and 4	22 (53.7 %)	20 (74.1 %)	2 (14.3 %)	
Unknown	2 (4.9 %)		2 (14.3 %)	
Comorbidities				
Diabetes	6 (14.4 %)	2 (7.4 %)	4 (28.6 %)	0.16 ${}^{b}$
Renal function ${}^{e}$				0.08 ${}^{c}$
KDIGO 1	17 (41.5 %)	12 (44.4 %)	5 (35.7 %)	
KDIGO 2	13 (31.7 %)	11 (40.7 %)	2 (14.3 %)	
KDIGO 3a	6 (14.6 %)	4 (14.8 %)	2 (14.3 %)	
NA	5 (12.2 %)	0	5 (35.7 %)	
Therapeutic immunosuppression ${}^{d}$	5 (12.3 %)	4 (14.8 %)	1 (7.1 %)	0.64 ${}^{b}$
Fixation of index prosthesis				0.62 ${}^{b}$
Uncemented	29 (70.7 %)	18 (66.7 %)	11 (78.6 %)	
Hybrid	10 (24.4 %)	7 (25.9 %)	3 (21.4 %)	
Cemented	2 (4.9 %)	2 (7.4 %)	0	
Surgical approach DAIR				0.09 ${}^{b}$
Anterior	31 (75.6 %)	21 (77.8 %)	10 (71.4 %)	
Anterolateral	1 (2.4 %)	1 (3.7 %)		
Lateral	3 (7.3 %)		3 (21.4 %)	
Posterior	3 (7.3 %)	2 (7.4 %)	1 (7.1 %)	
Trochanteric osteotomy/transfracture	3 (7.3 %)	3 (11.1 %)		
Quantities of CaSO ${}_{4}$ (Osteoset^®^ RBK 25 mL), n (%)				
1 pack	2	2		
2 packs	11	11		
3 packs	11	11		
4 packs	1	1		
Quantities of vancomycin, n (%)				
2000 mg	1	1		
4000 mg	10	10		
6000 mg	11	11		
8000 mg	1	1		
Quantities of ceftriaxone, n (%)				
2000 mg	1	1		
4000 mg	1	1		
Quantities of Osteoset^®^ T (10 mL), n (%)				
1 pack	1	1		
2 packs	1	1		

NA: not available. BMI is the body mass index. Results from ${}^{a}$ a chi-squared test, ${}^{b}$ Fisher's exact test, and ${}^{c}$ Wilcoxon's rank sum test. ${}^{d}$ Treatment includes systemic glucocorticoids and other medical immunosuppressive drugs such as methotrexate. ${}^{e}$ The glomerular filtration rate was estimated with the Cockcroft–Gault formula (Cockcroft and Gault, 1976) and was categorized analogous to the stages defined by KDIGO (kidney disease: improving global outcomes). The glomerular filtration rate could not be estimated for five patients in the group without AB-CaSO ${}_{4}$ as no indication of their weight was available. The serum creatinine values were within the normal range in these patients, though.

**Table 2:** Overview of the microorganisms identified in our study population. There were no statistically significant differences between the groups. The identification procedure for microorganisms changed during the study period. Until December 2017, only a phenotypical identification was performed. Since January 2018, matrix-assisted laser desorption/ionization time-of-flight (MALDI-TOF) spectrometry has allowed the identification of individual species. As more than one microorganism may be identified in a patient's samples, the total numbers do not add up. In total, four cases had a polymicrobial infection, i.e., two in each treatment group. Any identified microorganism had to fulfill the usual diagnostic criteria to be considered as causative (Zimmerli et al., 2004; Osmon et al., 2013). In two cases, culture-negative infections were treated. In both cases, clinical and radiological signs were present, indicating an overt infection. However, in these cases, antibiotic treatment had been started before sampling in a referring institution.

Microorganism	All patients ( n=41 )	DAIR with AB-CaSO ${}_{4}$ ( n=27 )	DAIR without AB-CaSO ${}_{4}$ ( n=14 )
*Staphylococcus aureus*	11 (26.8 %)	5 (18.5 %)	6 (42.9 %)
Coagulase-negative staphylococci	11 (26.8 %)	10 (37.0 %)	1 (7.1 %)
*Streptococcus* spp.	11 (26.8 %)	6 (22.2 %)	5 (35.7 %)
*Enterococcus* spp.	3 (7.3 %)	2 (7.4 %)	1 (7.1 %)
*Corynebacterium* spp.	2 (4.9 %)	1 (3.7 %)	1 (7.1 %)
*Propionibacterium* spp.	2 (4.9 %)	1 (3.7 %)	1 (7.1 %)
*Bacillus* spp.	1 (2.4 %)	1 (3.7 %)	
*Escherichia coli*	3 (7.3 %)	2 (7.4 %)	1 (7.1 %)
*Citrobacter * spp.	1 (2.4 %)	1 (3.7 %)	
*Pseudomonas aeruginosa*	3 (7.3 %)	2 (7.4 %)	1 (7.1 %)
Culture negative	2 (2.4 %)	2 (7.4 %)	
Polymicrobial	4 (9.7 %)	2 (7.4 %)	2 (14.3 %)

## 3 Results

Among the 41 patients treated using the DAIR procedure for PJI after THA with standard implants used for primary operations, 14 were treated between 2010 and 2017 without local application of antibiotics, and 27 were treated between 2015 and 2018 with AB-CaSO ${}_{4}$ . There were no planned second looks in the AB-CaSO ${}_{4}$ group, but there were three in the group without AB-CaSO ${}_{4}$ . The patient demographics and surgical data are summarized in Table 1. There were no significant differences between both groups, except for the ASA (American Society of Anesthesiology) score, with a higher proportion of healthier (ASA score 1 and 2) patients in the group without AB-CaSO ${}_{4}$ ( p=0.004 ). The distribution of cases of the study period is illustrated in Fig. 2. The spectrum of microorganisms identified from intraoperative sampling is shown in Table 2. All antibiotic treatments followed current international guidelines regarding the selection of drug and duration of administration (Zimmerli et al., 2004; Osmon et al., 2013). No patient received any long-term suppressive antibiotic treatment.

Considering any reoperation as outcome parameter, 11 of 14 cases treated without AB-CaSO ${}_{4}$ failed (79 %), whereas 4 of the 27 cases treated with AB-CaSO ${}_{4}$ failed (15 %). When considering the revision with component exchange as failure, these numbers were 9 out of 14 cases treated without AB-CaSO ${}_{4}$ (64 %) and, respectively, 4 of the 27 cases treated with AB-CaSO ${}_{4}$ (15 %). The Kaplan–Meier survival analysis showed that AB-CaSO ${}_{4}$ led to a significantly longer infection-free survival. The success rate regarding any reoperation ( p<0.0001 ; Fig. 3), and revision with component exchange ( p=0.0015 ; Fig. 4), was also in favor of the DAIR procedure with AB-CaSO ${}_{4}$ compared to standard DAIR. For all reoperations, the Cox proportional hazard ratio over the whole study period was 8.9 (95 % CI 2.8–28.2), whereas it was 5.6 (95 % CI 1.7–18.2) for the revision with component exchange, with both procedures favoring the addition of AB-CaSO ${}_{4}$ .

## 4 Discussion

In this study, infection-free survival and the success rates of DAIR for hip PJI greatly improved when AB-CaSO ${}_{4}$ was added to the standard care (Figs. 3 and 4; Zimmerli et al., 2004; Osmon et al., 2013). The differences in the survival curves over time were highly significant ( p<0.0001 ) when any reoperation was considered and remained significant ( p=0.0015 ) when only revisions with exchange of the prosthesis were considered as treatment failure. This second analysis was performed as repeated DAIR may be successful, and the recurrence of infection after a first DAIR does not necessarily require the removal of the implants (Wouthuyzen-Bakker et al., 2020). None of the patients received suppressive antibiotic treatment, which might have biased the indication for reoperation. The findings of this study were consistent, whichever outcome was analyzed. Expressed differently, the hazard ratio over the whole study period was 8.9 (95 % CI 2.8–28.2) for the outcome any reoperation, whereas it was 5.6 (95 % CI 1.7–18.2) for a revision with component exchange, with both procedures favoring the addition of AB-CaSO ${}_{4}$ despite the fact that the group treated without AB-CaSO ${}_{4}$ had a higher proportion of healthier patients, based on the ASA score (Table 1).

Our observations are in contrast to previous publications, which did not identify the advantages for the local delivery of antibiotics in DAIR (Flierl et al., 2017; Wouthuyzen-Bakker et al., 2019; Abosala and Ali, 2020). This may be due to a variety of reasons. While PMMA remains the standard carrier for local antibiotic delivery, drug elution kinetics are mostly unfavorable (Mueller et al., 2004; Hsieh et al., 2006; Anagnostakos et al., 2009; Wall et al., 2021). Aminoglycosides with bone cement serving as the carrier material may even be associated with worse outcomes in DAIR (Wouthuyzen-Bakker et al., 2019); this is not only due to the requirement of reoperation to remove the non-resorbable carrier (McKee et al., 2010). The choice of applied antibiotics may be another explanation. Aminoglycosides (gentamicin and tobramycin) are small molecules with a high solubility (DiCicco et al., 2003). When not covalently bound, these drugs rapidly elute from any carrier material, with the consecutive potential for systemic toxicity (Swieringa et al., 2008; Anagnostakos et al., 2009; Wahl et al., 2011; Overstreet et al., 2015). In addition, the mode of action of the aminoglycosides may be inadequate for orthopedic indications, as intracellular penetration that is necessary for the primary mode of action of inhibition of the protein synthesis is an oxygen-dependent active transport which is reduced in an acidic environment (Taber et al., 1987; Kadurugamuwa et al., 1993). In this case series, aminoglycosides were only added in two cases (Table 1) and with the intention of optimizing the effect of vancomycin or ceftriaxone, which are both known to have favorable elution kinetics from CaSO ${}_{4}$ (Wahl et al., 2017, 2018). The larger volumes of beads used in this case series, compared to the 10 mL of Stimulan^®^ (Biocomposites; Keele, UK) reported in a study with low success rates in DAIR for PJI after THA (Flierl et al., 2017), which is roughly equivalent to 25 mL Osteoset^®^, may also lead to a more favorable elution due to a bulk effect of the carrier material. Elution kinetics may also differ due to differences in the concentration of vancomycin and of tobramycin and explain the differences in the results (Flierl et al., 2017).

The current study has several limitations which have to be discussed. Patient care followed standard guidelines regarding patient selection and systemic antibiotic treatment (Zimmerli et al., 2004; Osmon et al., 2013). A rather small team of attending surgeons and infectious diseases specialists was involved, which ensured a certain standardization and continuity. Whereas the principles of the DAIR procedure were applied during the whole study period, the quality of the procedure may vary. The inclusion period was set to begin in 2010, as this corresponds to a switch in our department to the anterior approach as the standard approach in primary THA. However, CaSO ${}_{4}$ as the carrier material for the local delivery of antibiotics was introduced in our department only in 2015 (Fig. 2). The introduction of AB-CaSO ${}_{4}$ is associated with the raising of attention about PJIs by the team involved. However, we cannot comment on the significance of the impact of this increased awareness, but it may have to be considered as a potential bias. AB-CaSO ${}_{4}$ became the standard of care; the last DAIR without AB-CaSO ${}_{4}$ at our institution was performed in January 2017 (Fig. 1). From a methodological point of view, this represents only a short period of overlap. Basically, the current study compares two different treatment modalities performed in separate periods. Another limitation is the data quality of the retrospective patient file analysis. Thus, precise and detailed reconstruction might not have been possible for all cases. This is one of the reasons why DAIR repeated within 72 h was not considered as a failure but rather as a second look, which is a procedure commonly performed in the early study period. This ceased with the local application of AB-CaSO ${}_{4}$ . The success rate in the DAIR group without local antibiotic treatment appears to be rather low compared to other case series (Lora-Tamayo et al., 2013; Grammatopoulos et al., 2017b; Kunutsor et al., 2018; Wouthuyzen-Bakker et al., 2019; Shohat et al., 2020; Wouthuyzen-Bakker et al., 2020). Small numbers in the group without local antibiotics, mainly due to a high early failure rate, lead to a consecutively broad confidence interval in the later follow-up. Nevertheless, highly significant improvements of results were observed in the AB-CaSO ${}_{4}$ group. Our results for both DAIR with and without local antibiotic treatment with CaSO ${}_{4}$ appear similar to a recently published study with similar design (historical comparison) about knee PJIs (Gramlich et al., 2020). Only cases with regular implants for primary THA were included, limiting the generalizability, as larger revision or tumor implants are associated with worse outcomes (Wouthuyzen-Bakker et al., 2019). This may be due to a larger surface of the implants exposed to contamination, reduced possibilities to obtain radical debridement, or due to impaired soft tissues in complex reconstructions.

While corresponding to the spectrum of bacteria usually encountered in hip PJIs (Fulkerson et al., 2006; Moran et al., 2007; Schafer et al., 2008; Holleyman et al., 2016; Flierl et al., 2017; Gramlich et al., 2020), the microbiology in this case series was heterogeneous (Table 2). It is well known that the outcome for certain microorganisms is worse than for others (Lora-Tamayo et al., 2013, 2017; Wouthuyzen-Bakker et al., 2019; Shohat et al., 2020). Nevertheless, the spectrum of microorganisms may not explain the differences in results (Table 2). Failure happened nearly exclusively within the first few months after DAIR. This is in accordance with results from other studies investigating virulent species like streptococci and methicillin-resistant staphylococci (Lora-Tamayo et al., 2013; Flierl et al., 2017; Lora-Tamayo et al., 2017). Extending antibiotic coverage to gram-negative bacteria was only seldom necessary (Table 2). Standard systemic treatment always was 12 weeks, following the regimes usually recommended (Zimmerli et al., 2004; Osmon et al., 2013). Antibiotic treatment was not extended, as recommended recently for streptococci (Renz et al., 2019). No patients included in this study had long-term suppressive antibiotic treatment.

Curing infection is only one relevant issue among other parameters. Improving outcomes of DAIR procedures is very important, as functional outcomes for successful DAIR are much better than after exchange procedures are performed, particularly if staged (Dzaja et al., 2015; Grammatopoulos et al., 2017a; Herman et al., 2017). Reported success rates of DAIR, regarding the eradication of infection, vary from 35 % to 88 % (Deirmengian et al., 2003; Trebse et al., 2005; Marculescu et al., 2006; Vilchez et al., 2011; Font-Vizcarra et al., 2012; Aboltins et al., 2013; Lora-Tamayo et al., 2013; Flierl et al., 2017; Grammatopoulos et al., 2017b; Lora-Tamayo et al., 2017). In the group without local antibiotic application, the mid-term success rate was comparatively low, which points, of course, to other issues. On the other hand, the success of DAIR with AB-CaSO ${}_{4}$ has provided reliable results. None of the patients received antibiotic suppressive treatment, which would have biased the chosen outcome of reoperation. As the risk of recurrence becomes neglectable after some years of follow-up (Slullitel et al., 2021), no later failure of our cases is to be expected. In order to avoid issues with the potential prolonged wound drainage reported in association with CaSO ${}_{4}$ (Abosala and Ali, 2020), subcutaneous suction drains were commonly left in place for 5 d or more. As these drains are not intra-articular, contamination by continuity from the skin should not be an issue (Sankar et al., 2004; Ponnusamy et al., 2012). Rifampin should, however, be started only once the wound and any drain port sites have become dry, which might be delayed (Achermann et al., 2013). One of the most important factors for the success of DAIR is that the implants are well fixed (Grammatopoulos et al., 2017b). Time is, of course, associated with the development or maturation of the biofilm, which has consecutively decreasing treatment success rates (Burger et al., 1991; Marculescu et al., 2006; Vilchez et al., 2011; Fehring et al., 2013; Lora-Tamayo et al., 2013; Grammatopoulos et al., 2017a; Narayanan et al., 2018). Prolonged exposure to antibiotics at high concentrations may, however, overcome and even eradicate the matured biofilm (Post et al., 2017; Baeza et al., 2019). The required thresholds may be obtained for vancomycin with CaSO ${}_{4}$ as the carrier material for approximately 2 weeks, with concentrations remaining above usual MIC (minimal inhibitory concentration) for approximately 2 months (Wahl et al., 2017). When vancomycin is, thus, applied locally, the delayed introduction of rifampin due to drain port sites should not be an issue. The elution of ceftriaxone may even be more favorable, but clinical data are still lacking (Wahl et al., 2018).

In conclusion, this study shows a major advantage for local antibiotic treatment with CaSO ${}_{4}$ as the carrier material in the outcome of DAIR procedures for hip PJI when used as an addition to the standard treatment. Considering the subgroups, this conclusion is mainly valid for vancomycin, which was chosen regardless of the gram-positive bacteria involved, when considering known pharmacokinetics. Our results are, similarly, dramatically favorable to those in a recently published study about knee PJIs (Gramlich et al., 2020).

## Data Availability

Raw data may be made available upon request to the corresponding author.

## References

[bib1.bib1] Aboltins C, Dowsey MM, Peel T, Lim WK, Parikh S, Stanley P, Choong PF (2013). Early prosthetic hip joint infection treated with debridement, prosthesis retention and biofilm-active antibiotics: functional outcomes, quality of life and complications. Intern Med J.

[bib1.bib2] Abosala A, Ali M (2020). The Use of Calcium Sulphate beads in Periprosthetic Joint Infection, a systematic review. J Bone Joint Infect.

[bib1.bib3] Achermann Y, Eigenmann K, Ledergerber B, Derksen L, Rafeiner P, Clauss M, Nuesch R, Zellweger C, Vogt M, Zimmerli W (2013). Factors associated with rifampin resistance in staphylococcal periprosthetic joint infections (PJI): a matched case-control study. Infection.

[bib1.bib4] Anagnostakos K, Wilmes P, Schmitt E, Kelm J (2009). Elution of gentamicin and vancomycin from polymethylmethacrylate beads and hip spacers in vivo. Acta Orthop.

[bib1.bib5] Baeza J, Cury MB, Fleischman A, Ferrando A, Fuertes M, Goswami K, Lidgren L, Linke P, Manrique J, Makar G, McLaren A, Moriarty TF, Ren Q, Vince K, Wahl P, Webb J, Winkler H, Witso E, Young S (2019). General Assembly, Prevention, Local Antimicrobials: Proceedings of International Consensus on Orthopedic Infections. J Arthroplasty.

[bib1.bib6] Barros LH, Barbosa TA, Esteves J, Abreu M, Soares D, Sousa R (2019). Early Debridement, antibiotics and implant retention (DAIR) in patients with suspected acute infection after hip or knee arthroplasty – safe, effective and without negative functional impact. J Bone Joint Infect.

[bib1.bib7] Barton CB, Wang DL, An Q, Brown TS, Callaghan JJ, Otero JE (2020). Two-Stage Exchange Arthroplasty for Periprosthetic Joint Infection Following Total Hip or Knee Arthroplasty Is Associated With High Attrition Rate and Mortality. J Arthroplasty.

[bib1.bib8] Berend KR, Lombardi Jr AV, Morris MJ, Bergeson AG, Adams JB, Sneller MA (2013). Two-stage treatment of hip periprosthetic joint infection is associated with a high rate of infection control but high mortality. Clin Orthop Relat Res.

[bib1.bib9] Browne JA, Cancienne JM, Novicoff WM, Werner BC (2017). Removal of an Infected Hip Arthroplasty Is a High-Risk Surgery: Putting Morbidity Into Context With Other Major Nonorthopedic Operations. J Arthroplasty.

[bib1.bib10] Burger RR, Basch T, Hopson CN (1991). Implant salvage in infected total knee arthroplasty. Clin Orthop Relat Res.

[bib1.bib11] Cockcroft DW, Gault MH (1976). Prediction of creatinine clearence from serum creatinine. Nephron.

[bib1.bib12] Cooper HJ, Della Valle CJ (2013). The two-stage standard in revision total hip replacement. Bone Joint J.

[bib1.bib13] Deirmengian C, Greenbaum J, Lotke PA, Booth Jr RE, Lonner JH (2003). Limited success with open debridement and retention of components in the treatment of acute Staphylococcus aureus infections after total knee arthroplasty. J Arthroplasty.

[bib1.bib14] DiCicco M, Duong T, Chu A, Jansen SA (2003). Tobramycin and gentamycin elution analysis between two in situ polymerizable orthopedic composites. J Biomed Mater Res B.

[bib1.bib15] Dzaja I, Howard J, Somerville L, Lanting B (2015). Functional outcomes of acutely infected knee arthroplasty: a comparison of different surgical treatment options. Can J Surg.

[bib1.bib16] Fehring TK, Odum SM, Berend KR, Jiranek WA, Parvizi J, Bozic KJ, Della Valle CJ, Gioe TJ (2013). Failure of irrigation and debridement for early postoperative periprosthetic infection. Clin Orthop Relat Res.

[bib1.bib17] Fischbacher A, Peltier K, Borens O (2018). Economic Analysis in a Diagnosis Related Groups System for Two-stage Exchange of Prosthetic-joint Infections. J Bone Joint Infect.

[bib1.bib18] Flierl MA, Culp BM, Okroj KT, Springer BD, Levine BR, Della Valle CJ (2017). Poor Outcomes of Irrigation and Debridement in Acute Periprosthetic Joint Infection With Antibiotic-Impregnated Calcium Sulfate Beads. J Arthroplasty.

[bib1.bib19] Font-Vizcarra L, Garcia S, Bori G, Martinez-Pastor JC, Zumbado A, Morata L, Mensa J, Soriano A (2012). Long-term results of acute prosthetic joint infection treated with debridement and prosthesis retention: a case-control study. Int J Artif Organs.

[bib1.bib20] Fulkerson E, Valle CJ, Wise B, Walsh M, Preston C, Di Cesare PE (2006). Antibiotic susceptibility of bacteria infecting total joint arthroplasty sites. J Bone Joint Surg Am.

[bib1.bib21] Gramlich Y, Johnson T, Kemmerer M, Walter G, Hoffmann R, Klug A (2020). Salvage procedure for chronic periprosthetic knee infection: the application of DAIR results in better remission rates and infection-free survivorship when used with topical degradable calcium-based antibiotics. Knee Surg Sports Traumatol Arthrosc.

[bib1.bib22] Grammatopoulos G, Bolduc ME, Atkins BL, Kendrick BJL, McLardy-Smith P, Murray DW, Gundle R, Taylor AH (2017). Functional outcome of debridement, antibiotics and implant retention in periprosthetic joint infection involving the hip: a case-control study. Bone Joint J.

[bib1.bib23] Grammatopoulos G, Kendrick B, McNally M, Athanasou NA, Atkins B, McLardy-Smith P, Taylor A, Gundle R (2017). Outcome Following Debridement, Antibiotics, and Implant Retention in Hip Periprosthetic Joint Infection-An 18-Year Experience. J Arthroplasty.

[bib1.bib24] Gundtoft PH, Overgaard S, Schonheyder HC, Moller JK, Kjaersgaard-Andersen P, Pedersen AB (2015). The “true” incidence of surgically treated deep prosthetic joint infection after 32,896 primary total hip arthroplasties: a prospective cohort study. Acta Orthop.

[bib1.bib25] Haddad FS, Ngu A, Negus JJ (2017). Prosthetic Joint Infections and Cost Analysis?. Adv Exp Med Biol.

[bib1.bib26] Herman BV, Nyland M, Somerville L, MacDonald SJ, Lanting BA, Howard JL (2017). Functional outcomes of infected hip arthroplasty: a comparison of different surgical treatment options. Hip Int.

[bib1.bib27] Heuberger R, Wahl P, Krieg J, Gautier E (2014). Low in vitro third-body wear on total hip prostheses induced by calcium sulphate used for local antibiotic therapy. Eur Cell Mater.

[bib1.bib28] Holleyman RJ, Baker PN, Charlett A, Gould K, Deehan DJ (2016). Analysis of causative microorganism in 248 primary hip arthroplasties revised for infection: a study using the NJR dataset. Hip Int.

[bib1.bib29] Hsieh PH, Chang YH, Chen SH, Ueng SW, Shih CH (2006). High concentration and bioactivity of vancomycin and aztreonam eluted from Simplex cement spacers in two-stage revision of infected hip implants: a study of 46 patients at an average follow-up of 107 days. J Orthop Res.

[bib1.bib30] Huotari K, Peltola M, Jamsen E (2015). The incidence of late prosthetic joint infections: a registry-based study of 112,708 primary hip and knee replacements. Acta Orthop.

[bib1.bib31] Kadurugamuwa JL, Clarke AJ, Beveridge TJ (1993). Surface action of gentamicin on Pseudomonas aeruginosa. J Bacteriol.

[bib1.bib32] Kunutsor SK, Beswick AD, Whitehouse MR, Wylde V, Blom AW (2018). Debridement, antibiotics and implant retention for periprosthetic joint infections: A systematic review and meta-analysis of treatment outcomes. J Infect.

[bib1.bib33] Kurtz SM, Ong KL, Lau E, Bozic KJ, Berry D, Parvizi J (2010). Prosthetic joint infection risk after TKA in the Medicare population. Clin Orthop Relat Res.

[bib1.bib34] Kurtz SM, Lau E, Watson H, Schmier JK, Parvizi J (2012). Economic burden of periprosthetic joint infection in the United States. J Arthroplasty.

[bib1.bib35] Lora-Tamayo J, Murillo O, Iribarren JA, Soriano A, Sánchez-Somolinos M, Baraia-Etxaburu JM, Rico A, Palomino J, Rodríguez-Pardo D, Horcajada JP, Benito N, Bahamonde A, Granados A, del Toro MD, Cobo J, Riera M, Ramos A, Jover-Sáenz A, Ariza J, REIPI Group for the Study of Prosthetic Infection (2013). A large multicenter study of methicillin-susceptible and methicillin-resistant Staphylococcus aureus prosthetic joint infections managed with implant retention. Clin Infect Dis.

[bib1.bib36] Lora-Tamayo J, Senneville E, Ribera A, Bernard L, Dupon M, Zeller V, Li HK, Arvieux C, Clauss M, Uckay I, Vigante D, Ferry T, Iribarren JA, Peel TN, Sendi P, Miksic NG, Rodriguez-Pardo D, Del Toro MD, Fernandez-Sampedro M, Dapunt U, Huotari K, Davis JS, Palomino J, Neut D, Clark BM, Gottlieb T, Trebse R, Soriano A, Bahamonde A, Guio L, Rico A, Salles MJC, Pais MJG, Benito N, Riera M, Gomez L, Aboltins CA, Esteban J, Horcajada JP, O'Connell K, Ferrari M, Skaliczki G, Juan RS, Cobo J, Sanchez-Somolinos M, Ramos A, Giannitsioti E, Jover-Saenz A, Baraia-Etxaburu JM, Barbero JM, Choong PFM, Asseray N, Ansart S, Moal GL, Zimmerli W, Ariza J, Group of Investigators for Streptococcal Prosthetic Joint (2017). The Not-So-Good Prognosis of Streptococcal Periprosthetic Joint Infection Managed by Implant Retention: The Results of a Large Multicenter Study. Clin Infect Dis.

[bib1.bib37] Lum ZC, Natsuhara KM, Shelton TJ, Giordani M, Pereira GC, Meehan JP (2018). Mortality During Total Knee Periprosthetic Joint Infection. J Arthroplasty.

[bib1.bib38] Marculescu CE, Berbari EF, Hanssen AD, Steckelberg JM, Harmsen SW, Mandrekar JN, Osmon DR (2006). Outcome of prosthetic joint infections treated with debridement and retention of components. Clin Infect Dis.

[bib1.bib39] McKee MD, Li-Bland EA, Wild LM, Schemitsch EH (2010). A prospective, randomized clinical trial comparing an antibiotic-impregnated bioabsorbable bone substitute with standard antibiotic-impregnated cement beads in the treatment of chronic osteomyelitis and infected nonunion. J Orthop Trauma.

[bib1.bib40] Moran E, Masters S, Berendt AR, McLardy-Smith P, Byren I, Atkins BL (2007). Guiding empirical antibiotic therapy in orthopaedics: The microbiology of prosthetic joint infection managed by debridement, irrigation and prosthesis retention. J Infect.

[bib1.bib41] Mueller M, de la Pena A, Derendorf H (2004). Issues in pharmacokinetics and pharmacodynamics of anti-infective agents: kill curves versus MIC. Antimicrob Agents Chemother.

[bib1.bib42] Narayanan R, Anoushiravani AA, Elbuluk AM, Chen KK, Adler EM, Schwarzkopf R (2018). Irrigation and Debridement for Early Periprosthetic Knee Infection: Is It Effective?. J Arthroplasty.

[bib1.bib43] Natsuhara KM, Shelton TJ, Meehan JP, Lum ZC (2019). Mortality During Total Hip Periprosthetic Joint Infection. J Arthroplasty.

[bib1.bib44] Osmon DR, Berbari EF, Berendt AR, Lew D, Zimmerli W, Steckelberg JM, Rao N, Hanssen A, Wilson WR, Infectious Diseases Society of America (2013). Diagnosis and management of prosthetic joint infection: clinical practice guidelines by the Infectious Diseases Society of America. Clin Infect Dis.

[bib1.bib45] Oussedik SI, Dodd MB, Haddad FS (2010). Outcomes of revision total hip replacement for infection after grading according to a standard protocol. J Bone Joint Surg Br.

[bib1.bib46] Overstreet D, McLaren A, Calara F, Vernon B, McLemore R (2015). Local gentamicin delivery from resorbable viscous hydrogels is therapeutically effective. Clin Orthop Relat Res.

[bib1.bib47] Ponnusamy V, Venkatesh V, Curley A, Musonda P, Brown N, Tremlett C, Clarke P (2012). Segmental percutaneous central venous line cultures for diagnosis of catheter-related sepsis. Arch Dis Child Fetal Neonatal Ed.

[bib1.bib48] Post V, Wahl P, Richards RG, Moriarty TF (2017). Vancomycin displays time-dependent eradication of mature Staphylococcus aureus biofilms. J Orthop Res.

[bib1.bib49] Prendki V, Ferry T, Sergent P, Oziol E, Forestier E, Fraisse T, Tounes S, Ansart S, Gaillat J, Bayle S, Ruyer O, Borlot F, Le Falher G, Simorre B, Dauchy FA, Greffe S, Bauer T, Bell EN, Martha B, Martinot M, Froidure M, Buisson M, Waldner A, Lemaire X, Bosseray A, Maillet M, Charvet V, Barrelet A, Wyplosz B, Noaillon M, Denes E, Beretti E, Berlioz-Thibal M, Meyssonnier V, Fourniols E, Tliba L, Eden A, Jean M, Arvieux C, Guignery-Kadri K, Ronde-Oustau C, Hansmann Y, Belkacem A, Bouchand F, Gavazzi G, Herrmann F, Stirnemann J, Dinh A (2017). Prolonged suppressive antibiotic therapy for prosthetic joint infection in the elderly: a national multicentre cohort study. Eur J Clin Microbiol Infect Dis.

[bib1.bib50] Renz N, Rakow A, Muller M, Perka C, Trampuz A (2019). Long-term antimicrobial suppression prevents treatment failure of streptococcal periprosthetic joint infection. J Infect.

[bib1.bib51] Sandiford NA, Hutt JR, Kendoff DO, Mitchell PA, Citak M, Granger L (2020). Prolonged suppressive antibiotic therapy is successful in the management of prosthetic joint infection. Eur J Orthop Surg Traumatol.

[bib1.bib52] Sankar B, Ray P, Rai J (2004). Suction drain tip culture in orthopaedic surgery: a prospective study of 214 clean operations. Int Orthop.

[bib1.bib53] Schafer P, Fink B, Sandow D, Margull A, Berger I, Frommelt L (2008). Prolonged bacterial culture to identify late periprosthetic joint infection: a promising strategy. Clin Infect Dis.

[bib1.bib54] Schwartz AM, Farley KX, Guild GN, Bradbury Jr TL (2020). Projections and Epidemiology of Revision Hip and Knee Arthroplasty in the United States to 2030. J Arthroplasty.

[bib1.bib55] Shohat N, Goswami K, Tan TL, Yayac M, Soriano A, Sousa R, Wouthuyzen-Bakker M, Parvizi J, Infections ESGOIA, Northern Infection Network Joint Arthroplasty (NINJA) (2020). 2020 Frank Stinchfield Award: Identifying who will fail following irrigation and debridement for prosthetic joint infection. Bone Joint J.

[bib1.bib56] Slullitel PA, Onativia JI, Cima I, Zanotti G, Comba F, Piccaluga F, Buttaro MA (2021). Patients with no recurrence of infection five years after two-stage revision hip arthroplasty may be classified as periprosthetic infection "in remission". Bone Joint J.

[bib1.bib57] Sousa A, Carvalho A, Pereira C, Reis E, Santos AC, Abreu M, Soares D, Fragoso R, Ferreira S, Reis M, Sousa R (2018). Economic Impact of Prosthetic Joint Infection – an Evaluation Within the Portuguese National Health System. J Bone Joint Infect.

[bib1.bib58] Svensson K, Rolfson O, Naucler E, Lazarinis S, Skoldenberg O, Schilcher J, Johanson PE, Mohaddes M, Karrholm J (2020). Exchange of Modular Components Improves Success of Debridement, Antibiotics, and Implant Retention: An Observational Study of 575 Patients with Infection After Primary Total Hip Arthroplasty. JB JS Open Access.

[bib1.bib59] Swieringa AJ, Goosen JH, Jansman FG, Tulp NJ (2008). In vivo pharmacokinetics of a gentamicin-loaded collagen sponge in acute periprosthetic infection: serum values in 19 patients. Acta Orthop.

[bib1.bib60] Taber HW, Mueller JP, Miller PF, Arrow AS (1987). Bacterial uptake of aminoglycoside antibiotics. Microbiol Rev.

[bib1.bib61] Trebse R, Pisot V, Trampuz A (2005). Treatment of infected retained implants. J Bone Joint Surg Br.

[bib1.bib62] Vanhegan IS, Malik AK, Jayakumar P, Ul Islam S, Haddad FS (2012). A financial analysis of revision hip arthroplasty: the economic burden in relation to the national tariff. J Bone Joint Surg Br.

[bib1.bib63] Vilchez F, Martinez-Pastor JC, Garcia-Ramiro S, Bori G, Macule F, Sierra J, Font L, Mensa J, Soriano A (2011). Outcome and predictors of treatment failure in early post-surgical prosthetic joint infections due to Staphylococcus aureus treated with debridement. Clin Microbiol Infect.

[bib1.bib64] Wahl P, Livio F, Jacobi M, Gautier E, Buclin T (2011). Systemic exposure to tobramycin after local antibiotic treatment with calcium sulphate as carrier material. Arch Orthop Trauma Surg.

[bib1.bib65] Wahl P, Guidi M, Benninger E, Ronn K, Gautier E, Buclin T, Magnin JL, Livio F (2017). The levels of vancomycin in the blood and the wound after the local treatment of bone and soft-tissue infection with antibiotic-loaded calcium sulphate as carrier material. Bone Joint J.

[bib1.bib66] Wahl P, Rönn K, Bohner M, Decosterd LA, Meier C, Schläppi M, Festa S, Gautier E (2018). In vitro study of new combinations for local antibiotic therapy with calcium sulphate – Near constant release of ceftriaxone offers new treatment options. J Bone Joint Infect.

[bib1.bib67] Wall V, Nguyen TH, Nguyen N, Tran PA (2021). Controlling Antibiotic Release from Polymethylmethacrylate Bone Cement. Biomedicines.

[bib1.bib68] Webb JE, Schleck CD, Larson DR, Lewallen DG, Trousdale RT (2014). Mortality of elderly patients after two-stage reimplantation for total joint infection: a case-control study. J Arthroplasty.

[bib1.bib69] Wouthuyzen-Bakker M, Sebillotte M, Lomas J, Taylor A, Palomares EB, Murillo O, Parvizi J, Shohat N, Reinoso JC, Sanchez RE, Fernandez-Sampedro M, Senneville E, Huotari K, Barbero JM, Garcia-Canete J, Lora-Tamayo J, Ferrari MC, Vaznaisiene D, Yusuf E, Aboltins E, Trebse R, Salles MJ, Benito N, Vila A, Toro MDD, Kramer TS, Petersdorf S, Diaz-Brito V, Tufan ZK, Sanchez M, Arvieux C, Soriano A, ESCMID Study Group for Implant-Associated Infections (ESGIAI) (2019). Clinical outcome and risk factors for failure in late acute prosthetic joint infections treated with debridement and implant retention. J Infect.

[bib1.bib70] Wouthuyzen-Bakker M, Lowik CAM, Ploegmakers JJW, Knobben BAS, Dijkstra B, de Vries AJ, Mithoe G, Kampinga G, Zijlstra WP, Jutte PC, Northern Infection Network Joint Arthroplasty (NINJA) (2020). A Second Surgical Debridement for Acute Periprosthetic Joint Infections Should Not Be Discarded. J Arthroplasty.

[bib1.bib71] Zimmerli W, Trampuz A, Ochsner PE (2004). Prosthetic-joint infections. N Engl J Med.

